# Field Survey of Health Perception and Complaints of Pennsylvania Residents in the Marcellus Shale Region

**DOI:** 10.3390/ijerph110606517

**Published:** 2014-06-20

**Authors:** Pouné Saberi, Kathleen Joy Propert, Martha Powers, Edward Emmett, Judith Green-McKenzie

**Affiliations:** 1 Department of Emergency Medicine, Division of Occupational and Environmental Medicine, Center of Excellence in Environmental Toxicology, University of Pennsylvania Health Systems, One Convention Ave, 4 Penn Tower, Philadelphia, PA 19104, USA; E-Mail: Judith.McKenzie@uphs.upenn.edu; 2 Department of Biostatistics and Epidemiology, University of Pennsylvania Perelman School of Medicine, Philadelphia, PA, 19104, USA; E-Mail: propert@mail.med.upenn.edu; 3 Department of Earth and Environmental Science, University of Pennsylvania, Philadelphia, PA, 19104, USA; E-Mail: marthap@sas.upenn.edu; 4 Department of Emergency Medicine, Center of Excellence in Environmental Toxicology, University of Pennsylvania Perelman School of Medicine, Philadelphia, PA 19104, USA; E-Mail: emmetted@mail.med.upenn.edu

**Keywords:** unconventional natural gas development, hydraulic fracturing, Marcellus Shale, Pennsylvania, health perceptions, patient concerns, symptoms, medical records

## Abstract

Pennsylvania Marcellus Shale region residents have reported medical symptoms they believe are related to nearby Unconventional Natural Gas Development (UNGD). Associations between medical symptoms and UNGD have been minimally explored. The objective of this descriptive study is to explore whether shale region Pennsylvania residents perceive UNGD as a health concern and whether they attribute health symptoms to UNGD exposures. A questionnaire was administered to adult volunteers with medical complaints in a primary-care medical office in a county where UNGD was present. Participants were asked whether they were concerned about health effects from UNGD, and whether they attributed current symptoms to UNGD or to some other environmental exposure. There were 72 respondents; 22% perceived UNGD as a health concern and 13% attributed medical symptoms to UNGD exposures. Overall, 42% attributed one or more of their medical symptoms to environmental causes, of which UNGD was the most frequent. A medical record review conducted on six participants who attributed their medical symptoms to UNGD revealed that only one of these records documented both the symptoms in question and the attribution to UNGD. The results of this pilot study suggest that there is substantial concern about adverse health effects of UNGD among Pennsylvania Marcellus Shale residents, and that these concerns may not be adequately represented in medical records. Further efforts to determine the relationship between UNGD and health are recommended in order to address community concerns.

## 1. Introduction

The current technology to extract high volumes of natural gas and oil from the ground to produce energy relies on complex engineering that was not required in the past when such fossil fuel reservoirs were shallow and more accessible. The production cycle includes, but is not limited to, seismic testing to locate ideal well sites, well completions including directional drilling, hydraulic fracturing and extraction, and natural gas processing and transport via pipelines. Hydraulic fracturing of the Marcellus Shale formation relies on pumping as much as five million gallons of surface water under high pressure into a well. This creates fractures and open joints in the shale, allowing release of the natural gas. Approximately 0.5%–2% of the water volume can consist of various chemical additives including biocides and volatile organic compounds (VOCs) [1]. Proppants such as silica sand are injected into the shale fractures to keep them open. The term Unconventional Natural Gas Development (UNGD) will be used to describe the extraction and production process, recognizing that as this technology becomes more common, it may become established as a “conventional” method.

High volume hydraulic fracturing processes may contaminate ground water leading to pollution of residential well water supplies in rural Pennsylvania, which may be unrecognized as these supplies are not monitored or regulated [2]. Boyer *et al*. reported that 80% of water well owners in counties with natural gas drilling had concerns about their well water quality [3]. Migration of VOCs, including benzene and toluene, and other hazardous air pollutants (HAPs) into the atmosphere of surrounding communities has been documented in Texas and Colorado where UNGD is occurring [4,5]. This is important as the interaction of released VOCs with solar radiation in the atmosphere is reported to create ground level ozone [6]. The Marcellus Shale formation that underlies two-thirds of Pennsylvania and extends into some neighboring states is derived from prehistoric marine structures. Accordingly, the brine returning to the surface along with natural gas (produced water) as well as waste water (flow-back) release Normally Occurring Radioactive Materials (NORM), such as radon as well as concentrated levels of dissolved heavy metals [7]. UNGD necessitates a supporting infrastructure and as such has led to excessive truck traffic and increased motor vehicle accidents in these mostly rural areas [8]. In general, residents become subject to hazards of noise and vibration, increased air pollution from diesel exhaust and ultimately the catalysis of social disruption and a dramatic change in the way of life [9]. First implemented in the 1990s in the region of Texas overlying the Barnett Shale, the growth in UNGD has been rapid. Indeed, two decades later, in 2007, the Pennsylvania Department of Environmental Protection issued 117 permits for unconventional wells. This number rose exponentially to a total of 6,000 wells by 2012 [10]. Most of these wells have been located in the southwest and northeast sections of the state.

With these changes have come resident concerns as noted by focus groups, community surveys, and anecdotal reports. There are reports of complaints to local and state authorities by local residents regarding natural gas well releases into the air, some as a result of compressor station explosions and natural gas flares in proximity to homes, with some of the flares burning continuously for weeks [11]. Local residents have also expressed concern about the handling of drilling wastes, which are believed to contain radioactive isotopes of heavy metals, typically found in deep shale [12]. These stories are echoed by reports from federal agencies and articles published in popular media [13,14]. A 2010 Health Impact Assessment for UNGD in Garfield County, Colorado by the School of Public Health at the University of Colorado- Denver found residents had concerns about unknown chemicals, increased traffic, and increased accidents [15]. In 2011, Goldstein *et al*. pooled data from public opinion forums concerning UNGD held in Pennsylvania and found that health ranked second on the list of concerns expressed in Southwestern Pennsylvania [16]. Bamberger and Oswald have published 24 cases of hydraulic fracturing impacts on human and companion animal health from different states where UNGD is occurring, including Pennsylvania [17]. Finally, after interviewing 33 Pennsylvania residents from areas with UNGD, Ferrar *et al*. reported that most residents (82%) perceived health impacts [18]. 

## 2. Methods

We conducted an exploratory descriptive study in an area with intensive UNGD to determine whether patients attending a single primary-care clinic perceived their symptoms to be associated with natural gas activities. Study participation was voluntary. The Institutional Review Board at the University of Pennsylvania approved this study.

The study was conducted over one week (Monday through Friday) during a summer month in 2012. All patients presenting to this clinic during the study period were invited to participate. There were two interviewers, each using the same written script to explain the study to participants. Informed consent was obtained from each participant. HIPAA compliance documentation and a written explanation of the study were provided to the study volunteer. The pilot-tested, self-administered questionnaire was completed during the time that the study subject waited to see the physician. Each one was given the option of providing additional consent for review of their medical records. 

Questions ranged from information about participant demographics, current symptoms using a 29 item check-list (see Table 1), the date of onset of any reported symptoms, any reasons for that day’s visit to the physician, and the patient’s opinion as to whether there was an environmental cause for any symptom using the categories listed (see Table 2). The participant had the option of writing in and specifying their own environmental exposure under the category “Other Causes.” 

**Table 1:** List of Symptoms in Survey Questionnaire.

Symptom	
Rashes/skin irritation	Chest pain/tightness
Hair loss	Palpitations
Irritated eyes	Dizziness
Headaches	Trembling of hands
Muscle aches	Balance difficulty
Joint pain	Numbness/tingling/burning in hands/feet
Dental problems	Blood in stool
Bleeding from gums	Diarrhea
Nosebleeds	Vomiting or nausea
Burning of the nose and throat	Abdominal pain
Ringing in ear	Blood in urine
Frequent sinus problems	Anxiety
Ear pain	Sadness
Wheezing	Sleep difficulties
Shortness of breath	

**Table 2:** Reported Frequency of Environmental Reasons for Medical Problems Among 72 Study Participants.

	Natural Gas Activity	Antibiotics in Food	Aging from Free Radicals	High Tension Power lines	Living Near Highways	Other Causes
N	16	11	6	2	1	20
%	28.6	19.6	10.7	3.6	1.8	35.7

If an individual attributed current symptoms to UNGD and had given consent to medical record review, the lead author, a physician, reviewed the medical records, including those for the current visit and any consultant or emergency room reports. All instances in which there was documentation that the patient or physician had previously raised the possible relationship of symptoms to UNGD exposure were recorded.

Statistical analysis included the use of REDCap for survey data entry and Stata 13.0 for analysis [19,20]. Descriptive statistics were used including means with standard deviations or proportions with associated confidence intervals (CIs). ArcGIS was used to create the map to evaluate data from participants who consented to having their addresses mapped [21]. Gas well locations were obtained from the Pennsylvania Department of Environmental Protection website [22].

## 3. Results

Of the 159 people approached to take the survey, 72 (45%) completed it. The average participant age was 56.5 years, 58.3% were female, and 45.8% had at least a high school education or higher. The average time for residing at their current address was 23 years, and 88% resided in Bradford County, Pennsylvania. 

### 3.1. Perceptions of Causation

Thirty of the 72 participants (41.7%) responded that one or more environmental reasons caused at least one of their health problems. Table 2 displays the frequency with which each environmental reason was chosen among these 30 respondents. A respondent may have chosen more than one reason. “Natural gas activity” was the most frequently cited environmental reason, as chosen by 16 of 72 participants (22.2% [95% CI: 13.3%, 32.7%]). Participants wrote in twenty environmental reasons under “other causes;” these reasons were diverse and none appeared more than once. Examples of “other causes” proffered included: food/preservatives, plastics, stress, radio waves, allergens, pollution/industry and electronic devices. Nine of the 16 participants nominating “natural gas activity” attributed one or more specific current medical symptoms to UNGD. The symptoms so attributed are listed in Table 3. These categories are not mutually exclusive and some subjects reported more than one symptom.

**Table 3:** Symptoms Attributed by Participants to UNGD (*n =* 9).

Organ System	Symptom	Number of Positive Responses
Mental health	Sleeping difficulty	2
	Anxiety	1
Head, ears, throat	Ringing in ear(s)	1
	Sinus problems/infection	2
	Headaches	1
Neurological	Balance difficulty	1
	Trembling of hands	1
	Tingling of hands and feet	1
	Dizziness	1
	Seizures	1
Gastro-intestinal	Nausea	2
	Vomiting	1
	Diarrhea	1
	Stomach pain	1
Cardiovascular	Palpitations	1

### 3.2. Medical Record Review

Six of the nine participants attributing current symptoms to UNGD consented to a review of their medical records. In one case both the symptom reported by the patient on the survey questionnaire and the patient’s concern about UNGD had been documented. For two cases, the symptom reported on the questionnaire appeared in the medical records, but there was no corresponding mention of concern about UNGD as the possible cause. In the remaining three cases, neither the symptom nor attribution to UNGD reported on the questionnaire appeared in the primary care providers’ medical records, consultant reports or emergency room records.

### 3.3. Mapping

Fifty-three (73.6%) of the 72 respondents consented to having their residential location mapped. This included 13 of the 16 participants who had health concerns about UNGD. No clear pattern of clustering around UNGD operations was discernable. However, it is noteworthy that all 53 lived within two miles of a UNGD facility, presumably reflecting the density of UNGD operations [22].

## 4. Discussion

We found that 22% of patients visiting a primary care medical practice in an area where there was extensive UNGD activity expressed concern about the potential of UNGD to harm health, with 12.5% of patients (*n =* 9) believing that one or more of their current symptoms were attributable to UNGD. The attribution of environmental factors as a cause for health symptoms has been borne out in other studies. One such study investigated the relationship between health risk perception and the prevalence of self-reported symptoms attributed to electromagnetic fields (EMF) in the general Swedish population and found that 5% (95% CI: 4%, 6%) of the population attributed their symptoms to electromagnetic hypersensitivity while 53% (95% CI: 51%, 55%) worried about adverse health effects from EMF, even though they did not attribute any personal health symptoms to EMF [23]. In a review of pertinent literature, a study by Vrijheid concluded that the perception of environment leading to health problems is prevalent in residents living near landfill sites. However, Vrijheid noted that given the lack of direct exposure assessment, “it is difficult to conclude whether these symptoms are an effect of direct toxicologic action of chemicals present in waste sites, an effect of stress and fears related to the waste site, or an effect of reporting bias” [24]. 

There are some similarities between the symptoms attributed to UNGD by the patients we surveyed and other reports of UNGD-exposed subjects. A Health Impact Assessment for Garfield County, Colorado found self-reported health effects including eye irritation, breathing problems, coughing, and pneumonia [15]. Veterinarians documented health symptoms in owners of companion animals where the death or illness of the animal was attributed to UNGD [17]. The most common symptoms were upper respiratory in nature, including burning of the nose and throat, as well as burning of the eyes. Also reported were headaches, vomiting, diarrhea, rashes, and nosebleeds. Steinzer *et al*. [25] surveyed Pennsylvania residents who had complained to community organizations about UNGD and found complaints of throat irritation, sinus problems, and severe headaches. Using the Chemical Abstract Service Numbers and likely human health impacts to delineate organ systems that could be affected by exposure to chemicals from UNGD, Colburn *et al*. categorized chemicals used in the hydraulic fracturing process. She concluded that that 70% of the chemicals could cause skin, respiratory and gastrointestinal symptoms [26]. 

Three of our participants attributed anxiety or sleep disturbances to UNGD. Other studies have more generally reported the presence of mental health issues related to environmental factors, attributing these findings to more intangible factors such as subjects’ loss of control over their surroundings [27]. Our study did not enable us to discern the nature of the causal connection between UNGD and the perceived mental health symptom consequence. 

A group of participants without UNGD attributed health problems still expressed concern about future health impacts of UNGD. Others studies have also reported concerns about future effects of UNGD. Merkel *et al*. surveyed well water users in Pennsylvania and found that parents were concerned about potential contamination of tap water by natural gas drilling processes [28]. 

Our initial findings, from a small number of observations, indicate that medical record review is unlikely to portray the extent of citizen concern, or to reflect the extent to which patients perceive that UNGD exposures are responsible for their illnesses. There are several possible explanations for the differences observed between medical record review and questionnaire responses—reasons that may be provider-based, and reasons that may be patient-based. Patients may not have wanted to “bother” the doctor with their worries, or patients may expect the health care provider to volunteer the cause of the illness. Provider-based reasons could include that the provider is not skilled or comfortable in evaluating and treating an environmental concern, or may feel powerless in preventing the environmental cause [29]. There is literature establishing the lack of concurrence between medical records and patient reports in other areas of medicine [30,31].

A number of phenomena may have contributed to widespread community concerns in Pennsylvania around UNGD. There is no well-established clinical framework with which to evaluate or treat persons who believe their symptoms are due to UNGD. Other than the health impact assessment in Colorado, conducted after the adoption of this new technology, no health impact assessments were performed elsewhere [32]. Some state agencies, including the Pennsylvania Department of Health, do not currently have a system for tracking health complaints around UNGD and lack a well-developed infrastructure to provide meaningful help to residents, workers, and health care professionals [33]. Despite several high profile stories alleging health impact from UNGD there have been no comprehensive studies of cause and effect to establish whether or not there is a scientific basis for the claims [34]. 

Better information is needed to understand the relationships between UNGD and health. This will help practitioners respond appropriately to symptoms patients perceive as related to UNGD. An understanding of causation is also necessary to facilitate appropriate preventive practices and policy development. Careful impartial and objective medical evaluations together with environmental assessments of individuals perceiving health consequences from UNGD exposure could help determine the nature of any syndromes and the identity of the likely cause, whether it be physical, chemical, or psychological. Longitudinal epidemiologic studies of changes in the incidence or prevalence of disease as UNGD expands may help establish temporal relationships between UNGD and health. Studies should incorporate comprehensive environmental assessment data to account not only for direct hydraulic fracturing operations but also for traffic density, pollution, and social changes—phenomena resulting from UNGD. Detailed geographic mapping of exposures and health outcomes may be helpful, although our early experience suggests that the utility of mapping will be complicated because of the high density and ubiquity of wells in UNGD intensive areas. Issues in documentation and policy development are discussed further elsewhere [35]. 

Improved understanding of cause and effect relationships should fuel education for patients and health-care providers to help deal with perceptions of causation. Patients, residents, and workers with concerns about potential health effects from exposure to UNGD need to express these concerns to medical providers to facilitate constructive encounters. In turn, medical providers will need support in interacting with patients with health issues they consider associated with UNGD. Departments of Health in any state where UNGD is occurring would be in an ideal position to provide or facilitate this education and support. 

There are limitations to this study. It was conducted over a one week period during the summer, and it is possible there could have been an increase or decrease in UNGD during this period, thus leading to greater attribution of cause by the respondents. As it was a volunteer study, selection bias may be a factor, as those who felt that their symptoms are due to an environmental cause may have been more likely to choose to participate. This selection bias was limited as much as possible, since a group who presented to a primary-care medical office was studied rather than a convenience sample of community volunteers. This study was necessarily small due to time and funding constraints and thus remained primarily descriptive. Another potential limitation is that the study participants resided mostly in one county in Pennsylvania, which limits its generalizability to other shale territories in the state, as well as other states. However, the questionnaire was administered by two researchers following the same script in an effort to minimize response bias. The questionnaire was pilot-tested in an effort to ensure accuracy, uniform understanding of the questions, consistency, and efficiency of completion. 

## 5. Conclusions

Residents we surveyed in a Marcellus Shale region are concerned about health problems related to UNGD. Some residents attending a primary-care clinic in that area are experiencing symptoms that they attribute to UNGD. There is a need to pursue environmental, clinical and epidemiological studies to better understand associations among UNGD, medical outcomes, and residents’ perception of risk. This will make possible improved education for the public and health-care providers regarding UNGD and its potential effects on health. These efforts will likely necessitate comprehensive involvement and cooperation of state and federal agencies, as well as legislators, health care providers, and area residents.
